# Plasticity in the Human Visual Cortex: An Ophthalmology-Based Perspective

**DOI:** 10.1155/2013/568354

**Published:** 2013-09-25

**Authors:** Andreia Martins Rosa, Maria Fátima Silva, Sónia Ferreira, Joaquim Murta, Miguel Castelo-Branco

**Affiliations:** ^1^Departamento de Oftalmologia, Centro Hospitalar e Universitário de Coimbra, 3000 Coimbra, Portugal; ^2^Faculty of Medicine, University of Coimbra, Azinhaga de Santa Comba, 3000-548 Coimbra, Portugal; ^3^Visual Neuroscience Laboratory, IBILI, Faculty of Medicine, University of Coimbra, Azinhaga de Santa Comba, 3000-548 Coimbra, Portugal

## Abstract

Neuroplasticity refers to the ability of the brain to reorganize the function and structure of its connections in response to changes in the environment. Adult human visual cortex shows several manifestations of plasticity, such as perceptual learning and adaptation, working under the top-down influence of attention. Plasticity results from the interplay of several mechanisms, including the GABAergic system, epigenetic factors, mitochondrial activity, and structural remodeling of synaptic connectivity. There is also a downside of plasticity, that is, maladaptive plasticity, in which there are behavioral losses resulting from plasticity changes in the human brain. Understanding plasticity mechanisms could have major implications in the diagnosis and treatment of ocular diseases, such as retinal disorders, cataract and refractive surgery, amblyopia, and in the evaluation of surgical materials and techniques. Furthermore, eliciting plasticity could open new perspectives in the development of strategies that trigger plasticity for better medical and surgical outcomes.

## 1. Introduction

Attempts to improve visual acuity and quality of vision have included advances in visual outcomes evaluation, imaging techniques, and surgical techniques. However, even if we had the perfect method to correct the optics of the eye, our vision would still be determined by the retina-brain interaction. Vision involves perception and not only an optically perfect image. Neuroplasticity refers to the ability of the brain to reorganize the structure and function of its connections in response to the changing environment [1]. It is considered that the brain is plastic and neural networks are initially shaped by experience during the sensitive period and subsequently stabilized during normal development [2]. However, there is growing evidence that visual plasticity occurs not only during childhood, as traditionally considered, but also during all stages of life in response to changes in sensory experience [1]. Functional magnetic resonance imaging (fMRI) has opened an unprecedented opportunity for studying brain activity *in vivo* and thus for better understanding plasticity in the visual cortex [3]. Other methodologies, such as psychophysics and in particular electroencephalography (EEG) and transcranial magnetic stimulation (TMS) may also offer the opportunity to investigate human brain functioning and plasticity. However, because it combines noninvasiveness with high spatial resolution, MRI has become the preferred imaging technique for the characterization of spatial-function relations occurring in plasticity-driven processes [4]. Moreover, if combined with psychophysics, it is a very powerful tool. The focus on pharmacology is also justified by the substantial amount of research on molecular mechanisms and how they can be tackled by pharmacological approaches. 

Plasticity can have major implications in the treatment of ocular and cerebral diseases and in the evaluation of materials and surgical techniques (including refractive surgery, cataract surgery, and presbyopia correction). Furthermore, in rodent models, plasticity can be elicited by reducing intracortical inhibition through pharmacologic treatment with antidepressants, which opens new perspectives in developing therapeutic strategies that harness plasticity for better outcomes [5].

This review focuses on the visual plasticity in the adult human cortex and its role on several ophthalmologic problems. We have organized this review in four major questions in order to answer a main question: Can visual plasticity be used in the future as a tool to correct ophthalmologic problems? The four major questions are as follows. (1) Does visual plasticity occur in adults? (2) What forms of visual plasticity exist in the human cortex? (3) What is the biological background of visual plasticity? (4) What is the relevance of visual plasticity for ophthalmology?

## 2. Question ****1: Does Visual Plasticity Occur in Adults?

Neuroplasticity can be thought as the subtle but orchestrated dance between the brain and the environment [1]. It is the ability of the brain to be shaped by experience and, in turn, for this newly rewired brain to facilitate the embracing of new experiences [1]. Although plastic changes in the brain can occur at any time point in the life cycle, they occur with varying degrees of success [5]. It is known that an abnormal visual experience early in life, usually caused by strabismus, anisometropia, or congenital cataracts, causes amblyopia, an unilateral reduction of best corrected visual acuity that persists during the patient's life [5]. The explanation for these findings is that there are transient connections that go through a process of Hebbian competition in which stronger input signals are favoured and unused connections are pruned permanently [6]. In other words, Hebbian competition works during normal early development to tune the connections to visual cortical neurons, eliminating nonefficient inputs and balancing the input from the two eyes [6]. fMRI has shown that visual dysfunctions in amblyopia occur both within and beyond primary visual cortex (V1) including extrastriate and later specialized cortical areas (V4+/V8, lateral occipital complex) [5]. The connectivity of geniculate-striate and striate-extrastriate networks is reduced, and both feedforward and feedback interactions are affected equally [7]. This is in apparent agreement with the traditional view in which the visual system is assumed to be hard-wired long before adolescence. 

However, it has been shown that visual acuity can be improved in amblyopic adults through practicing a perceptual learning task (repeating a demanding visual task, such as contrast detection, to improve performance) [8–11]. The improvement of visual function persisted after treatment, showing that the learning was more than a temporary adaptation, thus providing evidence for cortical plasticity in human adults [8–11]. Improvement of visual function after a perceptual learning task was also demonstrated in participants with normal or corrected to normal visual acuity [12]. Plasticity has also been recently demonstrated following retinal ganglion cell functional and structural loss in carriers of a Leber's hereditary optic neuropathy mutation [13].

Video game playing with the amblyopic eye has also been shown to induce cortical plasticity and improve spatial vision in amblyopic adults [14], providing further evidence of plasticity in the adult visual system.

It is likely that some cortical connections are inhibited rather than pruned and that, for some visual functions, there is visual plasticity in adolescence and adulthood [1]. These functionally dormant connections appear to provide the substrate for rapid readaptation in adulthood [15]. For example, there are reports of improved vision in an adult's amblyopic eye after vision in the fellow good eye was lost, with changes occurring so rapidly in some cases that new connections are unlikely to have formed [6].

Scholz et al. in a study involving juggling, a complex motor skill requiring visuo-motor integration, found an increase in dorsomedial occipital gray matter density, likely corresponding to functional visual areas V3A and area V7 [16]. Because activity in this area is implicated in visuospatial imagery, the mentioned changes were attributed to the visualization of the movements and ball trajectories involved in juggling. This study provides evidence for training related structural changes in healthy adult human brain, and, more specifically, in a visual area. 

Thus, plastic changes have been seen in the adult human cortex not only in association with overt lesions but also in healthy individuals as a function of experience and training [17]. There is also evidence of a relation in old age between regional cortical shrinkage and increased task-related activation in neuroimaging, suggesting that losses in regional brain integrity drive functional reorganization that compensates/masks cognitive losses from the atrophy [17].

In conclusion, the majority of studies point to the existence of plasticity in adult human visual cortex in response to visual loss in one or both eyes, and there is also a role for visual cortical plasticity in the absence of visual loss. 

## 3. Question ****2: What Manifestations of Visual Plasticity Exist in the Human Visual Cortex?

Functional MRI studies have shown that perceptual learning and voluntary attention can bias visual selection and modulate neuronal response in human adult visual cortex [18]. Adaptation is a form of rapid plasticity and leads to strong perceptual effects. By enhancing the visual processing of relevant information and reducing processing of ignored or redundant stimuli, learning, attention, and adaptation shape the landscape of our visual experiences [18].

### 3.1. Perceptual Learning

A behavioural manifestation of plasticity in humans is the perceptual learning, a process in which practicing a challenging task repeatedly leads to significant and persistent improvements in visual performance over time [15]. The effects of perceptual learning have been well documented beyond the critical period of development in visually normal adults [5]. It has been reported that perceptual learning elicits plastic changes in the visual system, as shown by changes in V1 activation during fMRI [5]. To evaluate this form of plasticity, neural activity has been measured after participants were intensively trained in a visual task, such as texture discrimination and detecting stimuli orientation [19]. Retinotopic increase in blood oxygenation level-dependent signal (BOLD) response after learning provides empirical support that learning favours activity in the visual cortex in order to increase the discrimination of trained targets from background flankers [20]. The improvement that has occurred in adults as well as in juveniles is specific to the trained eye and develops only across multiple days of training [15]. Training can improve the discrimination of small differences in the offset of two lines (Vernier acuity) and the ability to discriminate orientation, segregate elements of the visual scene, and detect small differences in the depth of two targets [21]. The recruitment of larger assemblies of interconnected neurons or sharpening of cell sensitivity to relevant features of the trained stimulus may produce a higher total neural response associated with increased regionally specific BOLD response [19, 22]. Perceptual learning in the visual system appears to be mediated primarily by changes in the response strength or the tuning of individual neurons, rather than large-scale spatial reorganization of the cortical network as found in the auditory and somatosensory systems [15].

More recently, in line with the benefits of perceptual learning, video games have been shown to improve perception, visuomotor coordination, spatial cognition, and attention, illustrating how an action game play can reshape the adult brain [2, 23]. These plastic changes have been shown to be long lasting, remaining even 2 years after the end of intervention [23]. Action game play primarily targets top-down, attentional systems, possibly altering the excitatory/inhibition balance to allow heightened plasticity [23]. Indeed, it has been shown that complex stimuli are typically not presented at a single retinal location, so their learning is nonspecific to retinal locations and does therefore occur in higher brain areas [12, 24]. Top-down projections from the frontal eye field to visual area V4 can enhance stimulus-related activity, which emphasizes the importance of high level mechanisms [21, 25].

However, the existence of intrinsic plasticity in V1 is controversial, as revealed by difficulties in identifying low-level processes that are context independent, truly local, and not the indirect result of higher level modulation [26, 27]. Additionally, there is increasing evidence for generalization of perceptual learning in conditions previously shown to be specific, such as the training of a different task at a different location allowing the transfer of the feature learning to the second location [12, 28]. This suggests that perceptual learning does indeed involve higher nonretinotopic brain areas that enable location transfer [12, 28]. Not only the retinotopic early visual cortex, but also the nonretinotopic higher brain areas are involved in visual discrimination [29, 30]. Thus, visual perceptual learning seems to involve more than the visual cortex; that is, it involves nonretinotopic higher brain areas, engaged in attention and decision-making [12, 29, 30]. 

### 3.2. Attention

Perceptual learning shows a strong interaction with attention, indicating that it is under top-down control [21]. Attention is necessary for the consolidation of memory and virtually all other forms of learning. One of the consequences of learning is to release performance from attentional control, leading to an automatization of the task [21, 31, 32]. Therefore, it is important to consider the influence of attention when evaluating manifestations of visual plasticity such as perceptual learning.

When processing a visual scene, there are mechanisms for selecting relevant and filtering out irrelevant information [33]. This function is accomplished by the attentional system. Two basic sources determine attentional processing: attention driven by the saliency of a signal (bottom-up) and intentions of the observer, mostly directed by task demands, that guide the focus of attention (top-down) [33]. Although these top-down influences originate in the frontal lobe, they primarily modulate neural activation in striate and extrastriate visual areas [33]. fMRI studies have shown that attention can enhance the fMRI signal at early stages of visual processing, including the primary visual cortex [34]. Spatial attention seems not only to enhance processing at attended locations but also to suppress processing at nonattended locations [35]. When more attentional capacity is allocated at central fixation, there is a reduction of cortical activation for task irrelevant peripheral stimuli [36]. The attentional effect increases from V1 to V4, along the hierarchy of visual areas [37]. Top-down signals related to spatially directed attention may be generated by a network of areas in frontal and parietal cortices [38]. Sensory activity in the brain is modulated by attention, memory, and even the intention to act [39]. As an example, in experiences with monkeys, the baseline firing rate of neurons in lateral intraparietal area increases when the animal is working in a task in which it expects that a relevant visuospatial stimulus will appear [39]. Likewise, imaging studies have shown that attention modulates visual responsivity in the human brain [39]. The visual system modifies the retinal image so as to maximize its usefulness to the subject, often originating nonveridical percepts [40]. The visual system does not provide a copy of the external visual world; in contrast, it optimizes processing resources. Attention is an example of this perceptual optimization [41]. Visual attentional load also influences plasticity in the human motor cortex, suggesting that the top-down influence of attention on plasticity is a general feature of the adult human brain [42]. In sum, attention acts upon sensory signals at many levels to construct a selective representation of visual space [39].

### 3.3. Adaptation

Looking at a pattern for a short time typically decreases sensitivity to that pattern and results in a bias in the appearance of other patterns [43]. Ordinary visual adaptation is considered to occur with brief exposures and their consequent aftereffects [44, 45]. We will refer to the ordinary adaptation as short-term adaptation. However, a process of long-term adaptation can also be found. In this case, a causal effect may be permanent, and the changes may be given by the structural plasticity following learning processes.

In short-term adaptation, there are changes in sensitivity over short time intervals, ranging from milliseconds to minutes [43]. A classic example is light adaptation. Changes occur so rapidly that structural plasticity is not able to explain it. In long-term adaptation, there are sensitivity adjustments that occur during much longer times, from hours to weeks or even years [43]. These long-term adjustments have been described for color vision, contrast sensitivity, and perceived distortion (blur) [45–47]. For example, when the senescent crystalline lens is removed in cataract surgery, the changes in color appearance follow a very long time course and are not entirely normal even months after surgery [43]. Adaptation has also been shown to occur in natural visual environment, to stimuli that reflect the type of images that observers encounter in everyday viewing [43]. Many aspects of natural vision are routinely regulated by adaptation. Thus, the way we perceive colors, faces, and scenes is strongly dependent on the specific environments we are adapted to [43]. Adaptation also occurs when there are changes in the observer, rather than in the environment, because of eye injury, cataract surgery, or simply a new pair of glasses. For example, adaptation to long-term defocus (myopia and hyperopia) leads to improvements in visual acuity [48].

However, the relationship between adaptation and learning is not entirely clear. The visual system has a large variety of adjustments, and it is difficult to define adaptation in a way that it can be clearly distinguished from other forms of plasticity [43].

Perceptual learning usually produces improvements in discrimination, whereas adaptation is a more immediate loss in sensitivity after exposure to a stimulus [49–51]. Learning can be distinguished from adaptation because it mainly reflects changes in performance rather than in appearance and facilitation instead of suppression. It has a longer time course and changes how the visual system interprets neural signs and not the strength of those signals [51]. 

Like adaptation, learning can also change the appearance of patterns, and like learning, adaptation can facilitate some discriminations [43]. In fact, the process of adaptation itself might contain forms of learning [45]. With prolonged experience, adaptation is transferred to a long-term memory that can be instantly engaged or disengaged, leaving no aftereffects (the existence of an aftereffect is thought to indicate the presence of an adaptation process or a transient recalibration process) [45]. Both short- and long-term adaptation can occur from the blur resulting from the optics of the eye, including low- and high-order aberrations [52]. In addition, compensatory adjustments of adaptation tend to mask sensitivity losses that appear with disease, so that observers may not be aware of developing visual impairment [43]. Similarly, compensation for age related losses implies that the process of adaptation remains largely functional in the senescent visual system [43, 53, 54]. Thus, adaptation may be important for matching vision to the optical quality of the eye throughout life.

In conclusion, the manifestations of visual plasticity in the human visual cortex include perceptual learning and adaptation, under the influence of attention for resource optimization. These mechanisms are important not only to improve the treatment of ophthalmic disorders but also to understand the crosstalk between the optical system and the brain.

## 4. Question ****3: What Is the Biological Background of Neuroplasticity?

Two types of neuroplasticity can be distinguished, although their frontiers are not well defined: structural plasticity and synaptic or functional plasticity. Synaptic plasticity refers to changes in synaptic activity, leading to changes in synaptic efficacy and in behaviour [55]. Structural plasticity refers to changes in neuronal morphology (axons, dendrites, and dendritic spines), suppression and creation of synapses, and genesis of new neurons and neurites. 

Repetitive electrical stimulation of animal nerve fibers can induce an immediate and prolonged increase in synaptic transmission. This effect is called long-term potentiation (LTP) [56, 57]. In contrast, low-frequency stimulation typically induces long-term depression (LTD). These synaptic mechanisms play a role in many forms of learning and memory as well as neuronal development and circuit reorganization [57]. 

### 4.1. Physiological Mechanisms That Regulate Developmental Plasticity in the Visual System

Despite the fact that most of the mechanisms referred in the following paragraphs are active during the early phases of visual system development, we have included them in this review since some of them are being increasingly recognized as potential sources of plasticity reinstatement in the adult visual cortex.

The experience-dependent maturation of GABA-mediated inhibition during development establishes the beginning of the critical period for plasticity in the visual system [58]. After monocular deprivation during early life in transgenic animals lacking one isoform of GABA, no variation of visual cortex responsiveness was observed [59]. Therefore, a reduction of inhibitory transmission in early life halts the onset of the critical period for visual cortex plasticity [58]. The limited plasticity in the adult visual cortex can be enhanced by previous visual deprivation, which is associated with a loss of GABA receptors, and reduced by GABAergic modulators [60]. It has been shown that a brief reduction of GABAergic inhibition in the brains of rats is able to reopen a window of plasticity in the visual system a long time after the normal closure of the critical periods [5]. 

The effects caused by early sensory experience in the remodeling of visual cortical circuitries are preserved throughout life by the appearance of molecular factors in the extracellular milieu that restrict plasticity [61]. The establishment of neuronal connectivity may be, at least in part, under control of structural factors such as myelin-associated proteins (NgR, PirB) and chondroitin sulphate proteoglycans (CSPGs), which all are inhibitory for axonal sprouting [62]. Other important players are the major modulatory systems in the brain, that is, adrenaline, noradrenaline, dopamine, acetylcholine, and serotonin. The adrenergic system has a significant impact on plasticity [57]. Similarly, a single dose of the serotonin reuptake inhibitor citalopram enhances and prolongs plasticity [57]. Calcium channel blockade by nimodipine and dopamine receptor blockade by sulpiride or haloperidol diminish a form of plasticity [57]. Likewise, in the face of compromised cholinergic input to the visual cortex of rats, the ability to perform fine discriminations is impaired, whereas the ability to perform previously learned discrimination remains unaffected, which suggests that acetylcholine facilitates plastic changes in the sensory cortices [63, 64]. Functionally, acetylcholine contributes to plasticity in V1 and is involved in the alteration of tuning properties and map organization in other areas of cortex [64]. Global dopaminergic activation has heterogeneous effects on plasticity. A certain amount of activity of the dopaminergic system is necessary for the induction of plasticity. However, higher dopaminergic activity results in nonlinear effects on plasticity, depending on the dosage, the plasticity induction protocol, and the balance of D1 versus D2 receptor activation [57]. These mediators regulate complex functions of the central nervous system such as different forms of brain plasticity, cognitive processes, and behavior [62].

### 4.2. Functional Plasticity in the Visual Cortex

#### 4.2.1. Epigenetic Mechanisms of Plasticity, Short Noncoding mRNAs, and the Regulation of Plasticity

Growing experimental evidence indicates that chromatin structure is highly dynamic within the nervous system and that it is recruited as a target of plasticity-associated signal transduction pathways [62, 65, 66]. These mechanisms seem to be important also in the mature system, as increasing acetylation of histones by treatment with histone deacetylase inhibitors effectively reactivates plasticity in the adult visual system [67, 68].

Another mechanism involves CREB (a transcription factor) activity. CREB activity is induced following monocular deprivation in juveniles and declines with maturation of the visual cortex [69].

In addition to the function of transcription factors and modifications of chromatin structure, growing experimental evidence supports a critical role for short noncoding RNAs (microRNAs) which interact with and control translation of mRNA targets, in the regulation of gene expression patterns at the basis of plastic phenomena in the mammalian nervous system [70].

Experience-dependent brain plasticity is consolidated by sleep. This effect may be mediated through the phosphorylation of protein synthesis regulators and the translation of key plasticity-related mRNAs [71]. Sleep promotes cortical mRNA translation, and interruption of this process prevents the consolidation of a form of cortical plasticity *in vivo* [71]. This way, although experience is required for the transcription of key plasticity-related mRNAs, their translation into protein requires sleep, which may represent a sleep-dependent mechanism that converts labile plastic changes into more permanent forms [71].

#### 4.2.2. Mitochondrial Organization-Movement-Activity and Synaptic Activity

The brain can perceive, detect, discriminate, and recognize consciously only those pieces of information which reach a critical level, which can be at least indirectly related to bioenergetics and neuronal mitochondrial activity [72]. Representation of various sensory information can become conscious in our minds only if it reaches a threshold level of energy and duration [72].

Neurotransmitters dopamine and serotonin (which regulate different forms of brain plasticity, as explained previously) can reversibly control mitochondrial motility and distribution. Dopamine displays a net inhibitory effect on mitochondrial movement, but serotonin has a stimulatory effect [72]. There is a direct coupling between mitochondrial organization-movement-activity and synaptic activity [73].

Extension or movement of mitochondria into dendritic axons that are located far from the cell protrusions correlates with the development and morphological plasticity of dendritic spines [74, 75]. Molecular manipulations that reduce dendritic mitochondria lead to loss of synapses and dendritic spines [75]. In contrast, increasing dendritic mitochondrial content or mitochondrial activity enhances the number and plasticity of synapses [75]. This way, the dendritic distribution of mitochondria can be both essential and limiting for the support of synapses [13, 75, 76]. Moreover, mitochondrial gene upregulation has been observed following synaptic and neuronal activity [75].

Mitochondrial dysfunction leads to alterations in ATP production and cytoplasmatic calcium concentrations, reactive oxygen species, and nitric oxide production [77]. Mitochondria dysfunction has been implicated in the defective processes of plasticity occurring in schizophrenia [77].

Therefore, the spatiotemporal dynamic patterns of mitochondrial distribution can work as a “mitochondrial memory code” that dictates the potentiation of specific synapses and the plasticity of the neuronal network [78].

### 4.3. Structural Plasticity in the Visual Cortex

Animals under environmental enrichment (cages containing toys that are frequently changed) develop an increase in brain weight and cortical thickness, including the occipital cortex. Similarly, grey matter macrostructure changes have been reported in humans after juggling training, aerobic exercise, and intense language studies [16, 79–82]. Volume and thickness changes are specific to those brain regions that are functionally relevant for the trained task [83].

Neurochemical changes consisting of an increase in N-acetylaspartate (available almost only in neurons) were detected with magnetic resonance spectroscopy in adult men after a period of navigation training [79].

However, the existence of structural plasticity in the human primary visual cortex is controversial. It has been argued that both the location and apparent time course of structural changes vary substantially between studies, despite the similarity of the training paradigms [84]. Moreover, the reliability of voxel-based morphometry as a method for investigating structural brain changes has been questioned, as well as the biological substrate of the reported structural changes [84, 85]. In addition, studies involving cortical plasticity in the context of retinal lesions in humans have important limitations, as it is not possible to exclude spared retinal regions or changing borders in the absence of histological examination [85, 86]. It is also possible that V1 responses in the presence of central retinal lesions are due to activation via extrastriate cortex or subcortical structures [85, 87].

Despite the absence of large-scale structural remodeling later in life, the reorganization of cortical connections in terms of growth and loss of dendritic spines may be the structural substrate for experience-dependent plasticity [62].

**Figure 1:**
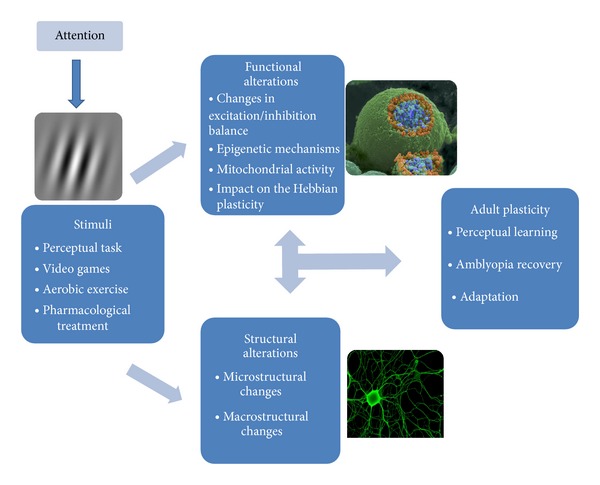
Plasticity in the adult visual cortex. In the presence of specific stimuli, such as performing a perceptual task, playing action video games, or pharmacological treatment, several functional alterations take place (image no. 214 from the Cell Image Library, neuron-neuron synaptic transmission). These include a decrease in inhibition/excitation ratio, epigenetic remodeling of chromatin structure, mitochondrial redistribution, activation of transcription factors, and protein synthesis. Structural plasticity includes modifications in neuronal morphology (axons, dendrites, and dendritic spines), suppression and creation of synapses, and genesis of new neurons and neuritis (image adapted from http://www.biomedcentral.com/1471-2202/6/24). The interplay of these mechanisms leads to adult neuronal plasticity, as revealed by the increased perception of a trained stimulus, improvement of visual function in amblyopia, and long-term adaptation to changes in the subject (such as cataract surgery) or in the environment. Plasticity is under the top-down influence of attention, as attention acts upon sensory signals at many levels to construct a selective representation of visual space.

In conclusion, the biological background of visual plasticity involves several mechanisms which are still incompletely characterized and controversial (Figure 1). Understanding these mechanisms will be important for a better recognition of the occurrence of plasticity and for disease treatment. As stated by Wandell and Smirnakis [85], it is not worth having a debate as to whether the brain is plastic or not: it is both. It is more important to study the conditions under which each system is stable or plastic.

## 5. Question ****4: What Is the Relevance of Visual Plasticity for Ophthalmology?

### 5.1. Plasticity in the Context of Retinal Disorders

Retinitis pigmentosa (RP) consists in an inherited progressive degeneration of photoreceptors, starting at the midperipheral (rod cells) and advancing towards the central retina (cone cells), with a subsequent deterioration of the retinal pigment epithelium. The absence or the segregation of mutated proteins provokes alterations in the regulated environment of rods. These cells undergo apoptosis, leading to a posterior degeneration of cones (it is believed that bipolar cells remain intact). The age of onset varies from infancy to adulthood, although the typical manifestations start at adolescence, making RP an appropriate way to study adult visual cortical plasticity [88, 89]. A recent fMRI study found visual cortical activation on the lesion projection zone (LPZ—region of visual cortex that is deprived of retinal input) [90] in striate areas of RP patients, during the performance of a visual task, in contrast with passive viewing stimulation. Authors suggested the unmasking of preexisting extrastriate feedback signals, which are blocked by lateral geniculate nucleus gating signals in the case of a healthy retina. However, the authors excluded the existence of large-scale reorganization, such as cortical rewiring or upregulation of existing synaptic connections [91, 92]. Another fMRI study showed crossmodal activity in the primary visual cortex of late-blind RP subjects during tactile tasks, while blindfolded. The authors also described a relationship between the level and the extension of cortical activation and the degree of vision loss in RP, suggesting adult cortical reorganization [93]. Parisi and coworkers used cortical visually evoked potentials to evaluate the relationship between retinal degeneration and visual cortical activation in RP individuals, encountering evidence of neural reorganization [94]. Wittich and colleagues described behavioral evidence of visual cortex plasticity, showing that RP patients have a similar ability to make spatial judgments as healthy subjects, despite the declining of their ocular function [92]. On the other hand, other studies did not report visual cortex reorganization in individuals with RP [95, 96].

Macular degeneration (MD), in contrast with RP, mainly affects the macula of the retina causing a progressive central vision loss. This disorder is associated with genetic mutations and environmental influences (aging, smoking, and diet). Wet MD is characterized by choroidal neovascularization that leads to an abnormal segregation of fluid or blood. Dry MD is more common and is caused by the accumulation of subretinal deposits and/or by hypo- or hyperpigmentation of retinal pigment epithelium. MD can affect elderly individuals—age-related macular degeneration (AMD)—or younger patients—juvenile macular degeneration (JMD). Thus, it is also a suitable model to analyze adult visual cortical plasticity [97]. Patients usually adopt a less stable peripheral retinal region for fixation without training or instruction—preferred retinal locus (PRL)—because the foveal region is absent due to a central scotoma. Some studies claim that this process results from primary visual cortex reorganization because deafferented neurons in LPZ become responsive to inputs near the retinal lesion, and the PRL is usually located in this area [98, 99]. An fMRI study identified activation of the LPZ with stimuli presented at the PRL or at another isoeccentric retinal location, indicating that reorganization is not driven by mechanisms related to the long-term use of PRL by patients, but it is instead a passive or spontaneous process [85, 98, 100]. Despite these results, the stimulation at PRL seems to be represented more extensively in visual cortex [101]. Another fMRI study presented different results where foveal cortex activation only exists for stimulation at PRL, correlating large-scale cortical reorganization and behavioral adaptations in MD. Authors proposed the enlargement of receptive fields, the strengthening of connections, extrastriate feedback, and/or changes in the network between visual areas and higher-order attention control areas as explanations for this reorganization [85, 99]. However, other authors did not report activation in the LPZ associated with the stimulation of the PRL [101, 102]. 

Another fMRI study with JMD and AMD patients indicated that visual stimulus falling on the peripheral retina activated the LPZ [85, 102]. Authors suggested the disinhibition/unmasking of intrinsic horizontal connections which spread activation from areas receiving retinal input to the LPZ, but this would require horizontal connections larger than normal V1 primate connections and polysynaptic chains of horizontal connections [102, 103]. They also proposed the growth of new horizontal connections, reorganization at precortical levels (but the previous literature reported absent or minimal reorganization at the lateral geniculate nucleus (LGN) and the retina), and top-down feedback from higher order visual areas associated with mental imagery and attention [85, 102–104]. In a later study, these authors concluded that this reorganization is only present for a completely loss of foveal vision, despite some possible local reorganization on the border of the LPZ [87, 103]. The level of cortical reorganization was not dependent on the age of onset or the type of MD [103]. Liu and colleagues demonstrated an incomplete functional reorganization where the extent of the LPZ was smaller in active than in passive viewing tasks. This effect was more prominent in JMD patients, suggesting a possible role of the age of onset and the disease etiology. Authors explained the results with the strengthening of feedback signals from higher cortical areas, associated with attention, during the task performance [101]. Masuda and colleagues found activation in the LPZ in JMD patients during an fMRI task related to the visual stimulus, but not during the stimulus passive visualization or a task unrelated to the stimulus [87]. They justified their results with the unmasking of task-dependent extrastriate feedback signals in the absence of input from the lateral geniculate nucleus, related to attention, visual imagery, and task-related low-level visual processing. Although similar results have been considered evidence of reorganization [101], these authors did not name it cortical reorganization, arguing that there was no change in neuronal architecture (synaptic gain or axonal connections) [85, 86].

Despite the evidence of functional reorganization in the adult visual cortex of JMD and/or AMD patients, some fMRI studies have questioned its existence [85, 105–107]. fMRI studies with simulated (artificial) central scotomata in healthy subjects presented enlarged and displaced receptive fields of cortical neurons, suggesting receptive field position, size scatter, and feedback signals from extrastriate cortex, thus questioning visual cortical reorganization [106, 108].

Kaas and colleagues' single-cell records in adult cats with induced central scotomata in one eye and enucleation of the other eye showed that neurons at the border of the LPZ responded to input from the area surrounding the retinal lesion. Receptive field sizes and response characteristics in LPZ were similar to normal cells after 2–6 months of visual deprivation, but receptive fields were displaced in LPZ. The authors proposed that this was due to “changes in the effectiveness of synapses within the arbors of thalamocortical axons of previously existing inputs” [85, 109–111]. Other studies with adult cats and adult monkeys with bilateral central retinal lesions demonstrated cortical retinotopic reorganization for stimulation outside the retinal lesion, due to the rapid expansion and shift of receptive fields of neurons near the border of the LPZ, some minutes after inducing scotomata, and due to long-range lateral cortical connections in the LPZ, after 2–12 months. However, the reorganization of LPZ was not complete, neuronal responses were weaker than normal, and the quality of orientation tuning of receptive fields was reduced [85, 104, 110, 111]. The short-term reorganization may be caused by the reweighting or the unmasking of existing neural connections. However, evidence for axonal and dendritic sprouting has also been identified in studies with adult cats as underlying mechanisms for long-term visual cortex reorganization [85, 87, 98, 99, 112].

 However, the existence of visual plasticity following retinal lesions in animal models remains controversial. Horton et al. found that cytochrome oxidase levels in V1 remained severely depressed even months after monocular retinal lesions in adult macaques [113]. Similarly, Murakami et al. found no evidence for topographic reorganization after monocular retinal lesions, using electrophysiological recordings. Smirnakis et al. used a 4.7-T fMRI in adult macaques to evaluate the existence of long-term cortical reorganization after retinal lesions [112]. There was no significant change in the position and size of the LPZ, which was also confirmed by electrophysiological measurements. The reason for these conflicting results may be due to retinal recovery after swelling caused by photocoagulation lasers used to induce scotomata, because researchers compare cortical responses after inducing the lesion to responses several months later. In addition, it is possible that reorganization might be restricted to some specific neurons inside the LPZ [112].

In conclusion, the degree of adult visual cortical plasticity due to retinal diseases remains questionable. However, the current view is that the visual cortex is plastic into the adulthood, although this plasticity is limited after the critical period [85, 114, 115]. 

Several hypotheses have been established to explain visual cortical reorganization: (1) development of synapses to create new lateral connections within V1, (2) large increase of synaptic signals that carry feedback and lateral connections, (3) unmasking of existing feedback signals from higher order cortical areas into V1 by deletion of feedforward signals, (4) increase of sizes and shift of receptive fields into the LPZ, and (5) modifications at precortical stages of visual system, although previous analyses suggested an absent or minimal reorganization at retinal and lateral geniculate nucleus levels [85, 87, 91, 101–104, 110]. It is known that feedback signals into primary visual cortex arise from higher order visual areas, frontal and parietal cortices, and are involved in attention, visual imagery, and task-related visual processing [85, 87, 91, 103]. Structural alterations of the adult visual cortex (establishment of new connections through dendritic growth, sprouting, and arborization) seem to be associated with a long duration of visual diseases, following rapid changes associated with modifications in the strength or the unmasking of preexisting connections. This can explain the “difference of reorganization between early- and late-blind individuals” [114].

The major limitations of studies concerning this issue are the reduced number of subjects, the heterogeneity among patients (nature of scotomata, disease duration, and progression), and the variations in methodologies (measurement of attentional state of subjects, monocular versus binocular stimulation, and delineation of the LPZ). In addition, there are difficulties in measuring the activity of the same neuronal cells before and after lesion and in establishing plasticity mechanisms with neuroimaging (scale of reorganization of several centimeters) versus electrophysiological techniques (scale of reorganization of few millimeters) [85, 87, 91, 92, 103, 111]. 

Future investigations are important to quantify the level of adult brain plasticity in visual processing. There is a lack of studies concerning the effects of peripheral vision loss on visual cortex (the majority of the literature presented above addressed central vision disorders) and the relationship between structural and functional visual cortical reorganization. 

### 5.2. Plasticity in the Context of Refractive Surgery

Anisometropia, a difference between the two eyes refractive errors generally exceeding 3 diopters, is an important cause of amblyopia. However, contrary to the expected persistency of visual deficiencies, refractive surgery (surgery that corrects refractive errors such as myopia, astigmatism, and hyperopia) is able to improve corrected visual acuity in amblyopic patients [116–118]. A study comparing fMRI activation patterns between preoperative and 12-month postoperative cortical maps found a decrease in the number of active voxels in the anisometropic fovea [119]. The proposed rationale for this finding was that before surgery a large network of neurons was activated for each visual stimulus. After surgery, however, only a subgroup of neurons is activated because stimuli processing has become more efficient [119]. This study thus provides evidence for plastic changes taking place in the primary visual cortex of adult anisometropic patients after refractive surgery and highlights the importance of visual plasticity even in the context of conditions requiring strictly ophthalmic procedures, such as refractive surgery.

### 5.3. Neuroadaptation to Presbyopia Correcting Intraocular Lenses

Presbyopia is the natural decline in near vision that occurs in human healthy aging. Surgical interventions to treat presbyopia and cataract are widely used, such as multifocal intraocular lenses, but they rely on the simultaneous presentation of distance and near images to the retina [120]. These lenses are associated with unwanted side effects, such as glare, halos, and loss of contrast sensitivity, that tend to improve over time in some patients, but not in others [121, 122]. These symptoms are usually more severe under low light (mesopic) conditions [123]. It is thought that the brain adapts to those unwanted stimuli, but it is unknown if it is an adaptive process or a form of perceptual learning. We hypothesize that multifocal intraocular lens may target different forms of plasticity, comprising (1) adaptation, triggered to decrease sensitivity to “background noise” images and glare, (2) perceptual learning, for better discrimination of low contrast targets, and (3) attention, to selectively see the image of interest despite the presence of two images (distance and near) in focus. Despite the fact that it is generally accepted that the brain plays a major role in visual performance with these more complex intraocular lenses, which is referred as *neuroadaptation*, there are no studies available evaluating cortical activity in the presence of multifocal lenses [124–127]. Strategies to increase plasticity mechanisms in the early postoperative period would likely improve the performance and comfort with these and other novel lenses.

### 5.4. Amblyopia Treatment and the Reinstatement of Plasticity in the Adult Visual System

Amblyopia can be considered the result of a lack of normal plasticity. Visual cortical dominance by the better eye leads to correspondent visual deprivation of the representations related to the eye with worse acuity. Knowledge of neuroplasticity and the factors that control the opening and closure of critical periods will lead to new therapeutic strategies which may allow for greater recovery of visual functions in both children and adults with amblyopia [5]. As previously described, the developmental maturation of intracortical inhibitory circuitries causes the end of plasticity in the visual system. In keeping with this notion, it is possible to restore plasticity in adult life by reducing levels of inhibition [5]. A direct demonstration that GABAergic signaling is a crucial brake limiting visual cortex plasticity was derived from the observation that a pharmacological decrease of inhibitory transmission effectively restores ocular dominance plasticity in adulthood [62]. Indeed, intracortical inhibitory circuitry has now emerged as a key factor in defining the limits of cortical plasticity [5]. It has thus been hypothesized that a critical factor in restoring plasticity and inducting recovery from amblyopia is to increase the ratio between excitation (glutamate receptors) and inhibition (GABA receptors) by reducing intracortical inhibition. In rodent models, plasticity can be elicited by reducing intracortical inhibition through pharmacologic treatment with administration of antidepressants [5, 67]. In humans, memantine, a glutamate receptor antagonist, abolishes a form of long-term potentiation plasticity. The GABAergic drugs diazepam, tiagabine, and baclofen also reduce this form of plasticity [57]. 

Amblyopia treatment has mainly involved performing perceptual learning tasks, such as contrast detection tasks with Gabor signals, as mentioned previously [8]. Improvement in visual acuity has been shown even when the training involves practicing a very different and functionally more basic task [8]. Similarly, Li et al. showed that after a brief period of video-game play a wide range of spatial vision functions improved substantially, reflecting normalization of visual acuity and positional acuity (low-level visual processing) and high-level processing (spatial attention, stereoacuity) [14].

### 5.5. Maladaptive Plasticity

In maladaptive plasticity there is a behavioral loss or the appearance of disease symptoms resulting from plasticity changes in the adult human brain [4]. 

Brain morphologic alterations in areas responsible for the transmission of pain were detected in patients suffering from different forms of pain, such as phantom pain, chronic back pain, neuropathic pain, irritable bowel syndrome, fibromyalgia, and headaches [128, 129].

Plasticity also underlies addiction-related processes, such as drug sensitization, drug seeking, and hypofrontality. Psychostimulant drugs such as amphetamine and cocaine are prototypic drugs inducing neuroplasticity changes [55].

Charles Bonnet syndrome can be thought as a form of maladaptive visual plasticity. This syndrome is characterized by complex, formed hallucinations occurring not only in patients without psychiatric disorders, usually after profound visual loss, but also in patients with visual field defects and normal central visual acuity [130–133]. Tan et al. proposed that after deafferentation caused by retinal or cortical lesions, the neurons become more responsive to neurotransmitter release by increasing the number and/or sensitivity of postsynaptic receptors [134]. Due to this increased sensitivity, normal levels of intracortical input trigger visual hallucinations. Because hallucinations tend to occur during visual recovery, the authors suggest that they are a correlate of visual system plasticity [134]. In this context, although they are usually a cause of concern, they may be a good prognostic sign, indicating the occurrence of neuroplasticity and visual field recovery [134]. 

Another downside of plasticity in the context of ophthalmology has been highlighted by Baseler et al. As the authors state, many of the most promising treatments for severe retinal disorders, such as prosthetics and stemcell therapy, rely on the assumption that the remaining cortical circuitry remains unchanged. This means that if it is possible to restore the input to the visual cortex, with novel retinal therapies, the neurons would be able to process this input effectively. Large scale plasticity could therefore jeopardize the effectiveness of these treatments.

## 6. General Conclusions

In conclusion, there are several forms of plasticity that remain largely functional in the adult visual system, as exemplified by perceptual learning and long-term adaptation. Both changes in the environment and in the observer are likely to involve different forms of plasticity that act together for perceptual optimization. Several biological systems are implicated in the interplay of functional and structural plasticity. Understanding how these mechanisms work could pave the way for new forms of diagnosis and treatment of ophthalmic disorders, comprising rehabilitation after severe retinal disorders, amblyopia treatment, and improvement of surgical results after cataract and refractive surgery.
